# Tracking epidemiological shifts in hepatitis A in Portugal: a comparison of seroprevalence between two nationwide surveys, 2001 to 2002 and 2015 to 2016

**DOI:** 10.2807/1560-7917.ES.2025.30.37.2500020

**Published:** 2025-09-18

**Authors:** Vera Manageiro, Rita Matos, Paula Palminha, Helena Cortes-Martins, Baltazar Nunes, Rita de Sousa

**Affiliations:** 1 Department of Infectious Diseases, National Institute of Health Doutor Ricardo Jorge (INSA), Lisbon, Portugal; 2 Department of Epidemiology, National Health Institute Doutor Ricardo Jorge (INSA), Lisbon, Portugal; 3 Comprehensive Health Research Center (CHRC), Centre for Public Health Research (CISP), National School of Public Health, NOVA University of Lisbon, Lisbon, Portugal

**Keywords:** Hepatitis A, seroprevalence, antibodies, Portugal, cohort effect

## Abstract

BACKGROUND: Hepatitis A incidence in Portugal declined from 20.1 to 0.4/100,000 population between 1987 and 2023, changing non-vaccinated population susceptibility. This shift has contributed to more frequent outbreaks, including in 2024–25, highlighting the need to enhance surveillance and integrate serological data.

AIM: We aimed to describe the exposure profile of the Portuguese population to hepatitis A virus (HAV) over time by estimating and comparing the seroprevalence of hepatitis A in two nationwide surveys.

METHODS: Data from two cross-sectional seroprevalence studies performed in 2001–02 and 2015–16 in the population aged ≥ 2 years were analysed. Seroprevalence was weighted for population distribution by age, sex and region, and then analysed by birth cohort (1911 -2014) and compared using Poisson regression.

RESULTS: Overall prevalence of anti-HAV IgG antibodies was 67.3% (95% CI: 64.2–70.3) in 2001–02 (n = 1,642) and 56.3% (95% CI: 52.4–60.2) in 2015–16 (n = 2,052), showing an 11-percentage-point decline. Birth cohort analysis revealed consistent seroprevalence within each cohort in both surveys, i.e. seroprevalence for the 1981–90 birth cohort was 16.7% and 18.7%, respectively, suggesting that higher seroprevalence is more closely associated with birth cohort (cohort effect) rather than a specific time point. Additionally, we found that individuals aged ≥ 30 years, born before the 1980s, and those with lower education had higher seroprevalence.

CONCLUSIONS: The immunological profile of anti-HAV antibodies in the Portuguese population has shifted over the last decades. High susceptibility and shifting age distribution of Hepatitis A-seropositive individuals highlight the need to revise future vaccination strategies in Portugal.

**Key public health message**

**What did you want to address in this study and why?**

Hepatitis A is an acute liver infection caused by hepatitis A virus (HAV) and transmitted primarily via the faecal–oral route, often through contaminated food or water. Portugal’s socioeconomic and hygiene improvements in the 1980s led to a reduction in HAV circulation and a decline in hepatitis A infections. However, this increased population susceptibility to infection. To measure immunity, we compared seroprevalence of HAV in the population and analysed changes over time.

**What have we learnt from this study?**

We found differences in HAV antibody prevalence between two population studies (2001–02 and 2015–16), mainly depending on individual’s birth year and their prior exposure to the virus. Specifically, we found that individuals aged < 30 years, as well as those born after the 1980s, were associated with lower HAV antibody prevalence. This indicates that more individuals are now susceptible to hepatitis A and that HAV is more likely to affect a wide range of age groups.

**What are the implications of your findings for public health?**

Unlike previous studies focusing on specific regions and/or groups, this study provides a nationwide overview of HAV immunity trends in the Portuguese population over time. Our study highlights that adapting vaccination strategies is important as more individuals become susceptible to hepatitis A. Thus, broad and early childhood vaccination can effectively reduce the spread of HAV.

## Introduction

Hepatitis A (HA) is the most common form of acute viral hepatitis in the world. Hepatitis A virus (HAV) is primarily transmitted via the faecal-oral route through ingestion of contaminated food and water, or through direct contact with a contagious person [1]. The clinical course of HA is age-dependent, ranging from asymptomatic, mainly in children under 5 years of age, to acute symptomatic hepatitis in older children and adults [2]. Symptoms range from mild to severe illness, involving prolonged jaundice and lengthy convalescence [2]. Although rare, HAV infection can cause acute liver failure and death, particularly in individuals aged over 50 years or with chronic liver disease [3]. The epidemiology of HAV infection depends on sanitation, socioeconomic conditions and, more recently, has been impacted by the introduction of HA vaccines (in 1998 in Portugal). In high-income countries, at-risk groups for HA include mainly international non-vaccinated travellers, men who have sex with men (MSM) and people who inject drugs [4-6].

Country levels of endemicity of HAV infection are assessed through the age-dependent prevalence of anti-HAV (IgG) antibodies in the population of the specific region [7]. According to World Health Organization (WHO) criteria, levels of endemicity have been classified based on seroprevalence as: high (≥ 90% by age 10 years); intermediate (≥ 50% by age 15 years, with < 90% by age 10 years); low (≥ 50% by age 30 years, with < 50% by age 15); and very low (< 50% by age 30 years) [7]. In countries with low or very low endemicity of HAV, almost all children and adults below the age of 40 years have not been exposed to HAV and remain susceptible to the infection [7]. Seroprevalence and susceptibility data of HA from European Union/European Economic Area (EU/EEA) countries showed that from 1975 to 1999, Portugal had intermediate HAV endemicity, similar to Cyprus, Greece, Italy, Romania and Poland [8]. However, improved sanitation over decades markedly reduced HAV circulation and infection rates (overall incidence 0.4/100,000 population in 2023), but also gradually increased population susceptibility, i.e. non-immune [9]. The increasing proportion of individuals susceptible to HAV infection, along with a shift in population exposure profile was indicated by some regional serological surveys and one nationwide study (published in 1984), aligning with seroprevalence trends observed in other European countries (Supplementary Table S1 provides a summary of seroprevalence studies in Portugal, 1984–2018) [10-16].

Although overall incidence rates remain low, countries with very low endemicity are increasingly susceptible to outbreaks, particularly within specific high-risk groups [4-6]. In Portugal, documented outbreaks involving a higher number (> 100) of cases have occurred in the Roma population (2004–05), the 2016–17 multi-country MSM outbreak, and two ongoing outbreaks (2023–25) affecting MSM and Roma communities [17-19]. Despite their distinct epidemiological profiles, with children being predominantly affected in the Roma outbreak and adults in the MSM outbreak, and different transmission patterns i.e. non-sexual vs sexual transmission respectively, these outbreaks clearly underscore the presence of a highly susceptible, non-immune population [17-19]. 

Continuous surveillance is essential for assessing population susceptibility and guiding public health interventions such as vaccination strategies. Additionally, strengthening surveillance systems and integrating serological and epidemiological data enhance outbreak preparedness and target prevention. To understand the nationwide exposure profile of the Portuguese population to HAV over time, our objective was to estimate and compare the HAV seroprevalence based on data from the two national serological surveys conducted in 2001–02 and 2015–16.

## Methods

### Study population and sampling method for serological surveys

This study included the data retrieved from two cross-sectional population-based serosurvey studies (Inquérito Serológico Nacional, ISN) carried out in 2001–02 and 2015–16 in the Portuguese population, whose results have been previously reported separately in national reports in Portuguese [20,21].

The target population in both surveys consisted of all individuals, aged 2 years and older, residing in Portugal for at least 12 months. Individuals were selected from those attending a national public or private laboratory to undergo a blood test for non-infectious diagnostic purposes, according to the established inclusion and exclusion criteria [20,21]. The testing involved examining a single sample from each individual and unique identifiers were assigned to ensure that each person's contribution was represented by only one sample.

In the ISN 2001–02, a stratified non-random scheme was used in which the strata were the age groups; the planned sample was subsequently allocated across the 18 districts of mainland Portugal in proportion to each district’s population. In the ISN 2015–16, the same non-random scheme stratified by age groups was used; however, the sample for each age group was allocated homogeneously among the seven Portuguese Nomenclature of Territorial Units for Statistics (NUTS) II (v. 2013) regions to obtain estimates with the same degree of precision. In both surveys, according to the study protocols, informed consent was obtained either from the participants themselves or from their legal representatives, and a blood sample was collected [20,21]. All data were irreversibly anonymised before analysis.

Raw databases containing the variables on population characteristics and serological results of the anonymous participants from both studies were submitted to recoding procedures, according to the questionnaire and inclusion criteria from each study, described in the corresponding reports [20,21]. Furthermore, data were combined in a new dataset to perform a joint analysis, using common variables, as described in Supplementary Table S2.

### Laboratory testing

Detection of anti-HAV-specific IgG antibodies in sera specimens was performed as previously described [18,19]. Comparable methodology was applied in both surveys, ensuring their internal validity and comparability. In ISN 2001–02, HAV-specific antibodies were determined using the HAVAB 2.0 (AxSYM, Abbott Diagnostics) kit/test, a qualitative sandwich microparticle enzyme immunoassay (MEIA) with reported 99.74% sensitivity and 98.96% specificity. In ISN 2015–16, the immunoassay was performed using the ARCHITECT HAVAb-IgG (Abbott Diagnostics) reagent kit/test a chemiluminescence microparticle (CMIA) immunoassay, with sensitivity > 98% and specificity 99.17%, according to the manufacturer’s information. The 2001–02 HAVAB 2.0 test measured total anti-HAV antibodies (IgM + IgG), while the 2015–16 ARCHITECT test measured only IgG; however, as samples were obtained from individuals undergoing diagnostic tests for non-infectious conditions, this methodological difference likely had minimal impact on the results. Reference sera were used to ensure reproducibility between surveys. In both ISN, samples with a signal-to-cut-off (S/CO) ratio of > 1.00 were considered (HAV seropositive).

### Statistical analysis

Descriptive statistics were used to compare the characteristics of the study population (age, sex, birth cohort, nationality, region of residence and education level) within and between each survey. For birth cohort analysis, participants were grouped according to decade of birth: 1911–20, 1921–30, 1931–40, 1941–50, 1951–60, 1961–70, 1971–80, 1981–90, 1991–2000, 2001–10 and 2011–14. At the time of the 2015–16 survey, the youngest cohort (2011–14) included individuals aged 2 years and older, reflecting the survey’s inclusion criteria. 

The HAV seroprevalence was estimated for the population as a whole and stratified by these birth cohorts and other demographic variables, after weighting for sex, age group and district of the Portuguese population in 2001 and 2015, for ISN 2001–02 and ISN 2015–16, respectively. This approach enabled us to assess the prevalence and distribution of those factors across the two survey periods while accounting for demographic changes over time. 

Poisson regression models with robust variance were used to estimate crude and sex‐ and age‐adjusted seroprevalence ratios (PR) and their respective confidence intervals of 95% (95% CI) as measures of association. The chi-square test was used to assess statistical significance and associations between study variables. Data were organised and analysed using Stata IC, v.16.1. (StataCorp LLC). All analyses were performed between September 2022 and September 2023.

## Results

A total of 1,642 (ISN 2001–02) and 2,052 (ISN 2015–16) individuals were included in the study after data cleaning. Supplementary Figure S1 provides a summary of the data cleaning process to create the final study population. The descriptive analysis of participants by age group, sex, birth cohort, education level, region of residence and nationality are described in the Table. Supplementary Table S2 provides the sociodemographic variables recoded and analysed in this study. The mean age was 34.4 (standard deviation (SD): 22.4) in 2001–02 and 28.2 (SD: 20.4) in 2015–16, of which 38.2% and 49.4% were male, respectively. Overall, the distribution of individuals by demographic and social characteristics in the two serosurveys were similar, with the exception that sampled participants were younger in ISN 2015–16. Supplementary Table S3 provides a comparison of age groups, sex, and region of residence across both surveys and with the Portuguese population in 2001 and 2015.

**Table:** Prevalence of anti-hepatitis A virus seropositivity and multivariate Poisson regression analysis by sociodemographic characteristics, Portugal, 2001–2002 (n = 1,642) and 2015–2016 (n = 2,052) serosurveys

Characteristics	ISN 2001–02 serosurvey								ISN 2015–16 serosurvey								PR (2015–16)/PR (2001–02)	95% CI
	n		Prevalence		Crude analysis		Adjusted analysis		n		Prevalence		Crude analysis		Adjusted analysis			
	Positive^a^	Total tested	%	95% CI	PR	95% CI	PR	95% CI	Positive^a^	Total tested	%	95% CI	PR	95% CI	PR	95% CI		
Total	951	1,642	67.3	64.2–70.3	NA		NA		725	2,052	56.3	52.4–60.2	NA		NA		0.84*	0.77–0.91
**Age group (years)**																		
< 30	224	820	31.0	26.1–36.3	Ref.		NA		204	1,287	11.4	9.1–14.3	Ref.		NA		0.37*	0.28–0.49
30–49	303	388	81.1	75.3–85.8	2.6	2.2–3.1			172	377	52.4	45.1–59.6	4.6	3.5–6.0			0.65*	0.55–0.75
≥ 50	424	434	97.9	95.1–99.1	3.2	2.7–3.7			349	388	93.9	90.4–96.2	8.2	6.5–10.3			0.96*	0.93–0.99
**Sex**																		
Male	360	627	66.6	61.5–71.4	Ref.		NA		364	1,014	54.4	48.8–60.0	Ref.		NA		0.82*	0.72–0.93
Female	591	1,015	67.9	63.9–71.6	1.0	0.9–1.1			361	1,038	58.1	52.6–63.3	1.1	0.9–1.2			0.86*	0.77–0.95
**Birth cohort (years)**																		
1911–1920	26	27	89.9	53.4–98.6	6.8	4.4–10.6	NA		0	0	NA		NA		NA		NA	
1921–1930	100	101	99.5	96.4–99.9	7.6	5.1–11.2			4	5	99.2	91.9–99.9	8.1	5.7–11.4			1.00	0.97–1.02
1931–1940	160	162	98.2	93.1–99.6	7.5	5.1–11.1			27	27	100.0	87.2-100.0	8.1	5.8–11.5			1.00	0.99–1.04
1941–1950	121	126	98.4	96.0–99.4	7.5	5.1–11.1			105	111	96.6	91.1–98.7	7.9	5.5–11.1			0.98	0.95–1.02
1951–1960	144	158	92.6	83.5–96.9	7.1	4.7–10.5			154	171	92.9	86.7–96.3	7.5	5.3–10.7			1.00	0.92–1.09
1961–1970	149	200	78.6	70.4–85.0	6.0	4.0–8.9			105	145	75.8	64.9–84.2	6.2	4.3–8.9			0.97	0.82–1.13
1971–1980	159	325	55.5	46.6–64.0	4.2	2.8–6.4			104	212	56.4	47.1–65.3	4.6	3.1–6.7			1.02	0.81–1.28
1981–1990	55	277	16.7	12.4–22.1	1.3	0.8–2.1			49	280	18.7	12.3–27.3	1.5	0.9–2.6			1.12	0.69–1.84
1991–2000	37	266	13.1	8.8–19.2	Ref.				89	475	12.3	8.6–17.2	Ref.				0.94	0.56–1.58
2001–2010	0	0	NA		NA				75	456	13.2	8.9–19.3	1.1	0.6–1.8			NA	
2011–2014	0	0	NA		NA				13	170	3.1	1.6–6.1	0.3	0.1–0.5			NA	
**Education level (≥ 25 years)**																		
No education/ Basic (1st cycle)	312	315	98.8	94.0–99.8	1.5	1.2–1.7	1.3	1.1–1.5	143	149	97.1	89.2–99.3	1.8	1.6–2.1	1.1	0.9–1.3	0.98	0.94–1.03
Basic (2nd cycle/3rd cycle)	155	193	84.5	76.6–90.1	1.2	1.0–1.5	1.2	1.0–1.5	154	219	79.8	71.2–86.3	1.5	1.3–1.8	1.3	1.1–1.5	0.94	0.84–1.07
Secondary	114	157	75.1	64.4–83.4	1.1	0.9–1.3	1.1	0.9–1.4	104	237	59.7	50.5–68.3	1.1	0.9 -1.4	1.2	1.0–1.4	0.79*	0.65–0.97
Higher education	116	179	68.0	57.1–77.3	Ref.		Ref.		142	339	53.0	44.9–60.9	Ref.		Ref.		0.78*	0.63–0.96
**Region of residence**																		
North	332	514	68.5	62.7–73.9	Ref.		Ref.		147	399	58.4	51.2–65.3	Ref.		Ref.		0.85*	0.74–0.99
Centre	253	435	68.9	63.5–73.8	1.0	0.9–1.1	0.9	0.9–1.0	122	379	58.3	51.3–64.9	1	0.8 -1.2	0.9	0.8–1.1	0.85*	0.74–0.97
Lisbon and Tagus Valley	303	553	64.6	58.8–70.0	0.9	0.8–1.1	0.9	0.8–1.0	222	563	52.0	45.0–59.0	0.9	0.7–1.1	0.8	0.7–0.9	0.81*	0.69–0.95
Alentejo	33	79	58.6	45.2–70.9	0.8	0.7–1.1	0.8	0.7–0.9	96	305	53.3	44.4–62.0	0.9	0.7–1.1	0.8	0.7–1.0	0.91	0.69–1.20
Algarve	30	61	62.4	48.4–74.5	0.9	0.7–1.1	0.8	0.7–1.0	138	406	57.4	51.4–63.2	1	0.8–1.2	0.9	0.8–1.1	0.92	0.73–1.17
**Nationality**																		
Portuguese	895	1,519	68.8	65.5–71.8	Ref.		Ref.		708	2,022	56.1	52.1–60.0	Ref.		Ref.		0.82*	0.75–0.89
Other	15	28	63.9	35.9–84.9	0.9	0.6–1.4	1.2	0.8–1.7	12	21	81.3	49.3–95.1	1.4	1.1–1.9	1.8	1.5–2.2	1.27	0.77–2.10
**Place of birth**																		
Portugal	Data not collected								637	1,837	56.3	52.1–60.4	Ref.		Ref.		NA	
South America									9	15	80.5	46.5–95.2	1.4	1.0–2.0	1.8	1.4–2.3		
Central and Eastern Europe									0	3	0		0		0			
Western Europe									7	32	43.0	18.0–72.2	0.8	0.4–1.6	1.1	0.6–2.0		
Southern Europe									0	2	0		0		0			
Africa									22	35	54.8	30.4–77.1	1.0	0.6–1.6	0.9	0.6–1.5		
Other									1	5	2.2	0.2–18.2	0.04	0.004–0.4	0.08	0.01–0.8		

CI: confidence interval; NA: not applicable; PR: prevalence ratio; Ref.: reference.

^a^ Number of individuals seropositive for anti-hepatitis A virus antibodies.

The ratio PR (2015–16)/PR (2001–02) with 95% CI shows the relative difference in prevalence between the two surveys. The chi-square test was used to assess statistical significance and associations between study variables. Significant differences (p < 0.05) are marked with an asterisk.

### Prevalence of anti-hepatitis A virus antibodies by age and birth cohort

The overall HAV seroprevalence was 67.3% (95% CI: 64.2–70.3) in the 2001–02 survey and 56.3% (95% CI: 52.4–60.2) in 2015–16, showing an absolute decrease of 11 percentage points between studies. Regarding age, we observed a decrease in the seroprevalence at age groups < 30 years: 31.0% (95% CI: 26.1–36.3) in 2001–02 compared with 11.4% (95% CI: 9.1–14.3) in 2015–16, representing a relative decrease of 63% (Table).

**Figure 1:**
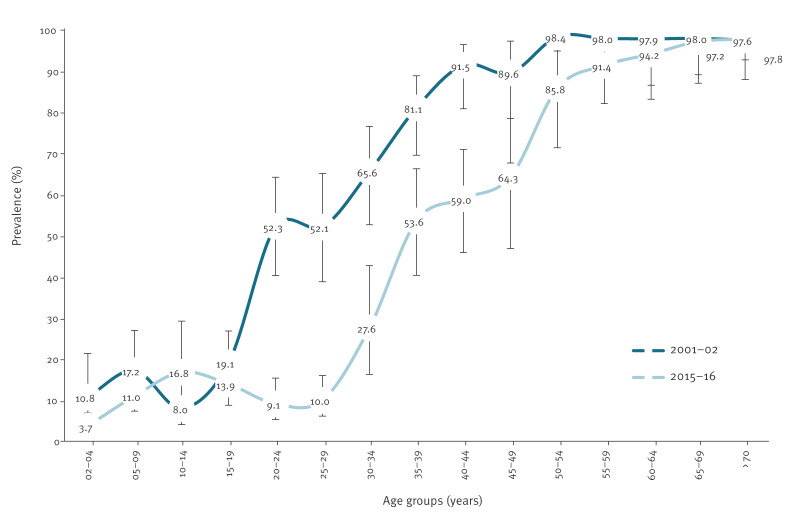
Age-dependent hepatitis A seroprevalence per 5-year age group, Portugal, 2001–02 (n = 1,642) and 2015–16 (n = 2,052) serosurveys

The estimated seroprevalence data described by 5-year age groups in the ISN 2001–02 and ISN 2015–16 are presented in Figure 1. Overall, HAV seroprevalence was higher in the first survey (2001–02). Indeed, we found a seroprevalence above 80% in the age groups 35–49 years in 2001–02, whereas in 2015–16 this level of seroprevalence was only observed in those aged ≥ 50 years. The only exception was for age group 10–14 years in which the seroprevalence was lower in 2001–02 (8.0; 95% CI: 4.1–14.9) compared with 2015–16 (16.8; 95% CI: 8.9–29.2), with a PR of 0.7 (95% CI: 0.3–1.9) and 4.6 (95% CI: 1.9–11.0), respectively (data not shown). Within each serosurvey, the prevalence of anti-HAV IgG antibodies was highest among those aged ≥ 50 years and clearly lower in the younger age groups (< 30 years).

The dark blue (2001–02) and light blue (2015–16) lines represent the prevalence estimates for each 5-year age group, with numbers indicating the observed percentages. Vertical bars show the 95% confidence intervals around each estimate.

**Figure 2:**
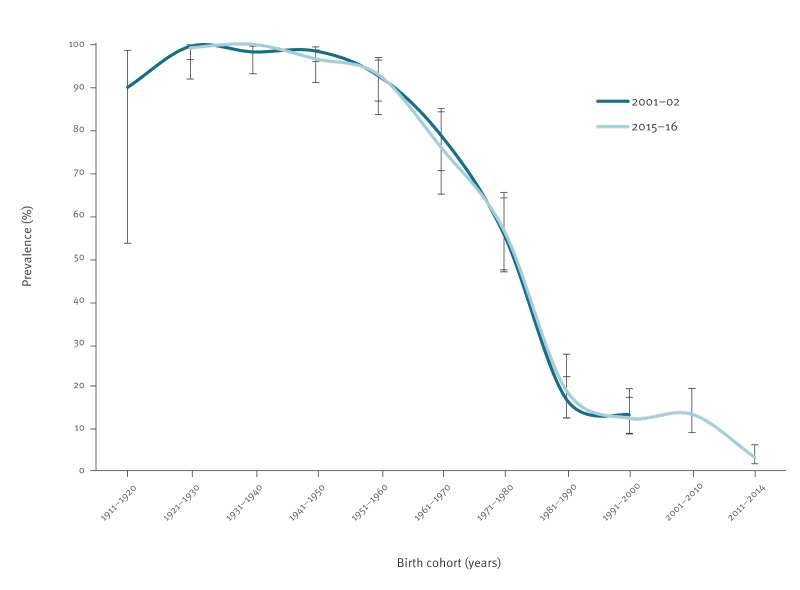
Birth cohort analysis of hepatitis A seroprevalence, Portugal, 2001–2002 (n = 1,642) and 2015–2016 (n = 2,052) serosurveys

The birth cohort analysis, using the defined groups (1911–20 to 2001–14), showed a decreasing trend in the prevalence of anti-HAV antibodies from the oldest to the more recent cohorts (Table, Figure 2). In the birth cohort 1981–90, the seroprevalence was 16.7% (95% CI: 12.4–22.1) in 2001–02 and 18.7% (95% CI: 12.3–27.3) in 2015–16, compared with 55.5% (95% CI: 46.6–64.0) and 56.4% (95% CI: 47.1–65.3) in the cohort 1971–80, respectively. This cohort effect can be observed in both Figures 1 and 2, where the consistent seroprevalence observed in one cohort during both the 2001–02 and 2015–16 surveys, e.g. for birth-cohort 1981–90 the seroprevalence was 16.7% and 18.7% respectively, implies that seroprevalence is associated more with the birth cohort (cohort effect) than with a specific time point.

The dark blue (2001–02) and light blue (2015–16) lines represent prevalence estimates for each birth cohort, with observed percentages shown. Vertical bars depict the 95% confidence intervals surrounding each estimate.

### Prevalence of anti-hepatitis A virus antibodies by sex, nationality, region of residence and education level

Seroprevalence was higher in the 2001–02 survey compared with the 2015–16 survey, regardless of sex, region of residence, nationality or education level (Table). Within each survey, the seroprevalence did not differ between males and females, or region of residence (Table). However, both studies showed similar education inequalities, with higher seroprevalence values among those with lower education. Indeed, in the 2015–16 serosurvey, the highest seroprevalence was associated with no education/basic (1st cycle) education level (97.1%; 95% CI: 89.2–99.3) and the lowest seroprevalence to higher education level (53.0%; 95% CI: 44.9–60.9). There was an 80% increase in the seroprevalence in the lowest education level (PR: 1.1; 95% CI: 0.9–1.3), when using higher education level as reference. Similar trends in the prevalence were seen in the 2001–02 serosurvey, with a 50% increase in the seroprevalence in the lowest education level (PR: 1.3; 95% CI: 1.1–1.5). Seroprevalence declined across all education levels between surveys with PR of 0.78 (95% CI: 0.63–0.96) for higher education and 0.98 (95% CI: 0.94–1.03) for no education, representing the change over time within each education level.

Regarding nationality, in the 2015–16 survey, being non-Portuguese was positively associated with higher prevalence of anti-HAV antibodies (adjusted PR: 1.8; 95% CI: 1.5–2.2), with an absolute increase of 17.4 percent points when compared with ISN 2001–02. Considering the birthplace (variable only available in the 2015–16 survey), the highest seroprevalence (80.5%; 95% CI: 46.5–95.2) was identified in those who were born in South America (Venezuela, Brazil), with an adjusted PR of 1.8 (95% CI: 1.4–2.3) with Portuguese birthplace as reference (Table). The individual countries specified for birthplace are provided in Supplementary Table S2. The average age of those born in South America was 35.8 years (95% CI: 27.3–44.3), with 88.9% being female (data not shown).

### Annual incidence rate

**Figure 3:**
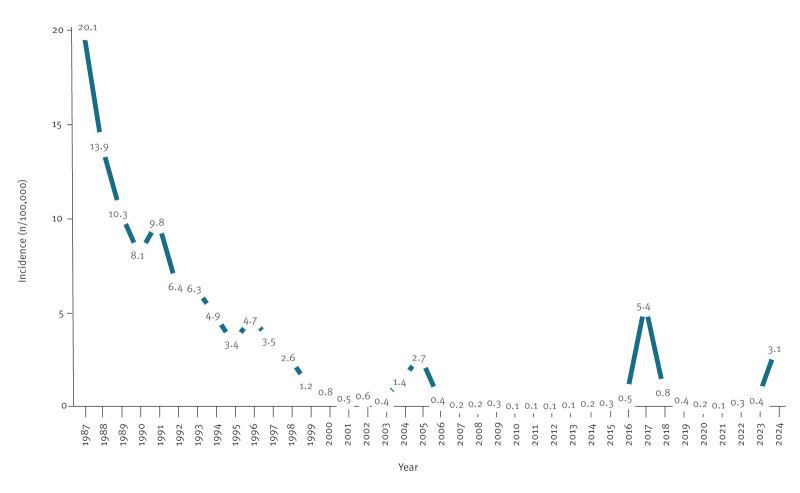
Annual incidence rate of hepatitis A cases, Portugal, 1987–2024 [9,17]

According to the number of cases notified per year, the cumulative incidence of HA in Portugal decreased from 20.1 per 100,000 population (n = 2017) in 1987, to 3.5 per 100,000 (n = 359) in 1997, and to 0.4 per 100,000 population (n = 40 cases) in 2023 (Figure 3).

The annual incidence rate of hepatitis A in Portugal from 1987 to 2024 is represented by a blue line, with the numbers indicating the observed rates per 100,000 population for a given year. Data are compiled from ECDC Surveillance Atlas of Infectious Diseases [9], and Portuguese Directorate-General for Health (DGS) Health Statistics Portal [17].

In 2024, the overall incidence in Portugal increased to 3.1 cases per 100,000 population (provisional data, pending official publication), corresponding to an absolute increase of 2.7 cases and an eightfold rise compared with 2023 (Figure 3). This marked increase is attributable to the ongoing outbreaks [17-19].

## Discussion

This study describes the nationwide profile of the exposure to HAV over time in the Portuguese population. Overall, a decrease in anti-HAV IgG antibodies prevalence was observed in the Portuguese population between the two analysed surveys, from 2001 to 2002 to 2015–16. Our findings indicate a possible cohort effect in the evolution of anti-HAV antibody prevalence between the two cross-sectional studies.

In our study, we observed a decline in HAV seroprevalence from 67.3% (95% CI: 64.2–70.3) in the 2001–02 survey to 56.3% (95% CI: 52.4–60.2) in 2015–16. This decrease in immunity was particularly evident among the younger populations. In 2001–02, seroprevalence exceeded 80% in the 35–39-year age group, whereas in 2015–16, similar seropositivity was only observed in individuals aged ≥ 50 years. Consequently, the proportion of adolescents and adults susceptible to HAV increased. These findings corroborate that Portugal transitioned from a low-endemicity country for HA in 2001–02 (≥ 50% by age 30 years, with < 50% by age 15), to a very low-endemicity country in 2015–16 (< 50% by age 30 years) [22].

Until the early 1970s, Portugal had very poor sanitary conditions, resulting in high mortality and morbidity caused by infectious diseases [23]. The political changes following the Carnation Revolution (Portuguese: Revolução dos Cravos) in April 1974, led to remarkable public sector investments, notably in water, sanitation and hygiene conditions, which likely contributed to the shift in HAV immunity among generations born after the 1980s. Our results show that individuals who were not immune in 2001–02 remained susceptible in 2015–16, suggesting a cohort effect associated with birth year and exposure history. Supporting this, a 1997 study on healthcare workers and medical students from a hospital in Lisbon showed a stable seroprevalence among those aged ≥ 30 years, while among workers under 30 years it was 65%, significantly higher than in students (< 46%) [24]. This emphasises a stronger link between seroprevalence and birth cohorts or occupational exposure rather than age alone. Similar epidemiological shifts have been observed across Europe, driven by improvements in hygiene and socioeconomic conditions [25]. In Finland, seroprevalence dropped below 40% in early 1940s, coinciding with the end of the Second World War [23]. Likewise, a Dutch study showed a lower seroprevalence in individuals born after the war, marking a turning point in hygiene standards [26]. While the transition to very low endemicity reflects improved living conditions, it has also led to increased susceptibility.

In our study, being under 30 years old and born after the 1980s was associated with lower prevalence of HAV antibodies, indicating a growing proportion of susceptible adolescents and adults over time in Portugal. The correlation between HAV prevalence and socioeconomic indicators observed in both serosurveys, such as higher prevalence of anti-HAV antibodies among individuals with no education/basic (1st cycle) or only basic education, is supported by other studies [12].

In the 2015–16 survey, non-Portuguese nationality and birth in South America were positively associated with higher HAV seroprevalence. Individuals from Venezuela and Brazil had an average age of 26.2 years, meaning they were born in the 1980s. Historically, South America was considered a high endemicity region, with most infections occurring in early childhood (≥ 90% of the population have acquired natural immunity by age 10). However, changes in HAV circulation patterns were also observed with a reported shift from high to intermediate endemicity, i.e. ≥ 50% by the age of 15 [27]. Improved socioeconomic conditions, along with the implementation of childhood immunisation programs in HAV endemic countries, have been effective in substantial decreases on the number of infections and increasing population immunity [22,27-29]. 

By early 2020, 28 countries worldwide had already introduced the HA vaccine into routine childhood immunisation programmes, leading to a decline in disease incidence [22,30]. This includes 10 countries in the Americas, five in the Eastern Mediterranean, eight in Europe and five in the Western Pacific [28,30]. In Portugal, the HA vaccine has only been available since 1998 as a monovalent formulation or combined with hepatitis B, however, it has not yet been included in the National Immunisation Programme and is only recommended for high-risk groups and travellers to endemic countries. This policy, combined with higher susceptibility among individuals, contributes to an increased proportion of travel-associated cases. Notably, in 2023, travel-related cases accounted for 34.2% of all reported HA cases [9]. In our study, the survey questionnaires did not include the vaccination status and vaccinated individuals were not excluded. Although some studies have shown that certain types of antibodies can be distinguished between naturally acquired infection and immunisation, commercial tests are not yet available [31]. 

In Portugal, the HA vaccine is recommended only for at-risk groups, leading to an anticipated low vaccination rate. The higher seroprevalence observed in the 2001–02 survey among the 5–9-year age group compared with the 10–14-year age group (Figure 1) might reflect localised differences or sampling variation rather than increased vaccination coverage, as this pattern was not consistent across all regions of Portugal. Indeed, there were no national HA vaccination campaigns for children, nor were there large outbreaks in the late 1990s that would have triggered widespread immunisation in this age group. In fact, regional studies conducted around that time, such as in Braga, confirmed low HAV immunity among young children and indicated that none of the participants had been vaccinated [12].

One limitation of our study was the use of non-random samples, with participants in both surveys being younger than the general Portuguese population, particularly in the ISN 2015–16. Since seroprevalence tends to increase with age, the younger age structure of our sample may have led to an underestimation of the true seroprevalence in the Portuguese population. However, the comparison remains valid as the same pattern was observed in both surveys. Furthermore, the 2001–02 sample had a higher female-to-male ratio than the Portuguese population, while the 2015–16 study included a greater proportion of the participants from Alentejo and Algarve, and fewer from the northern region. The 2001–02 survey’s sample was designed to reflect the total Portuguese population distribution at that time, based on demographic data and population densities, whereas the 2015–16 survey prioritised broad geographical representativeness, to address potential biases presented in the earlier survey. Despite differences in sample distribution between the two surveys, both datasets provide valuable insights into Portugal´s seroepidemiological trends over time. No measurement bias was detected, as laboratory methods and analytical procedures used to detect anti-HAV antibodies remained consistent across both surveys. This consistency ensures that any observed differences in seroprevalence are not due to variations in testing or measurement techniques but rather reflect true epidemiological changes in the population.

## Conclusions

The evolution of the anti-HAV immunological profile among the Portuguese population over the past decades underscores the need to re-evaluate HA vaccination strategies to address changing susceptibility trends. Large-scale early childhood vaccination and enhanced surveillance could help to mitigate transmission risks and guide target interventions. Aligning policies with international recommendations and adopting evidence-based strategies, Portugal can strengthen its efforts towards HA prevention and control, ultimately contributing to improved public health outcomes.

## Data Availability

Data are available upon reasonable request to the corresponding author.

## Supplementary Data

336000 Supplement
