# Supplementary material for: Tracking epidemiological shifts in hepatitis A in Portugal: a comparison of seroprevalence between two nationwide surveys, 2001 to 2002 and 2015 to 2016
Source: Euro Surveill. 2025 Sep 18;30(37):2500020. doi: 10.2807/1560-7917.ES.2025.30.37.2500020 (PMC12449678; doi:10.2807/1560-7917.ES.2025.30.37.2500020)
Supplement: Supplement [file 25-00020_DESOUSA_Supplement.pdf]

## Supplementary Material

This supplementary material is hosted by *Eurosurveillance* as supporting information alongside the article "**Tracking epidemiological shifts in hepatitis A in Portugal: a comparison of seroprevalence between two nationwide surveys 2001 to 2002 and 2015 to 2016**", on behalf of the authors, who remain responsible for the accuracy and appropriateness of the content. The same standards for ethics, copyright, attributions, and permissions as for the article apply. Supplements are not edited by *Eurosurveillance* and the journal is not responsible for the maintenance of any links or email addresses provided therein."

**Table S1.** Summary of seroprevalence data from previous studies by study year, population type and region, and sample size, Portugal, 1984-2018.

| Citation | Study Year | Population Type [region] | Sample Size (n) | Seroprevalence Data | |
|---|---|---|---|---|---|
| | | | | overall (%) | by age (%) |
| **Lecour, 1984 (1)** | 1983? | healthy individuals, continental Portugal | 1,770 | 84.9 | 23.6 (age 1-4); 61.3 (age 5-9); 76.4 (age 10-14); 93.4 (age 15-19); 96.5 (age 20-29); 99.2 (age 30-39); 99.0 (age 40-49); 99.1 (age ≥50) |
| **Lecour, 1986 (2)** | 4-year period [before 1985] | in-patients with acute viral hepatitis [Porto District] | 400 | 47.0 | 91.1 (age <15); 10.9 (age ≥15) |
| **Leitão, 1996 (3)** | - | Individuals living in urban (n=308) and rural (n=386) areas [Coimbra District] | 667 | 81.4 | - |
| **Marinho, 1997 (4)** | 1990-1992 | medical students (MS) and healthcare workers (HCW) [Lisbon District] | 526 | 86.4 (HCW) 35.3 (MS) | 29 (age <20) – MS; 48/65 (age 20-29) - MS/HCW; 69 (age 30-39) – HCW; 81 (age 40-49) – HCW; 77 (age ≥50) - HCW |
| **Macedo, 1998 (5)** | 1993 | asymptomatic healthy volunteer blood donors [Porto District] | 300 | - | 95 (age 18-25); 99 (age 26-45); 99 (age ≥46) |
| **Barros, 1999 (6)** | - | randomly sampled school children [Porto District] | 667 | 27.9 | 20.9 (age 6-7); 22.6 (age 8-9); 25.4 (age 10-11); 36.1 (age 12-13); 34.1 (age 14-15); 30.8 (age 16-17); 37.8 (age 18-19) |
| **Lecour, 1999 (7)** | - | healthy teenagers (n=1161) and adults (n=681) [North Region] | 2,042 | 29,7 (teenagers) 71.6 (adults) | - |
| **Cunha, 2001 (8)** | 1996 | outpatients without acute hepatitis [Braga District] | 381 | - | 7.0 (age 1-4); 15.8 (age 5-9); 26.9 (age 10-14); 51.2 (age 15-19); 85.5 (age 20-29); 72.5 (age 30-39); 87.8 (age 40-49); 88.7 (age ≥50) |
| **Antunes, 2004 (9)** | 1999 (age 14) 2000-2001 (age 5) 2002- | healthy children ≤14 years old [Braga District] | 536 | - | 1.6 (age 5); 3.9 (age 8); 32.5 (age 14) |

| | | | | | |
|---|---|---|---|---|---|
| | 2003 (age 8) | | | | |
| **ISN 2004 (10)** | 2000-2001 | All individuals resident in Portugal mainland for at least 12 months, ≥2 years-old, that attend clinical analysis labs | 1,665 | 57.5 | 9.9 (age 1-4); 20.0 (age 5-9); 9.9 (age 10-14); 22.6 (age 15-19); 38.8 (age 20-24); 53.6 (age 25-29); 59.3 (age 30-34); 79.0 (age 35-39); 90.5 (age 40-44); 5.6 (age 45-49); 5.6 (age 50-54); 5.3 (age 55-59); 8.2 (age 60-64); 98.8 (age ≥65) |
| **Valadas, 2008 (11)** | - | HIV-infected adults, hepatitis A unvaccinated population [Lisbon District] | - | 77.0 | 33.3 (age 20-29); 65.8 (age 30-39); 76.7 (age 40-49); 93.5 (age ≥50) |
| **Antunes, 2009 (12)** | 2005-2006 | Portuguese pregnant women that attend the hospital [Braga District] | 669 | 79.4 | 35.7 (age <20); 72.6 (age 20-29); 92.5 (age ≥30) |
| **Speidel, 2010 (13)** | 2006-2009 | Patients admitted to the hospital [Coimbra District] | 935 | 66.3 | 35.0 (age 20-29); 61.9 (age 30-39); 87.3 (age 40-49); 98.7 (age ≥50) |
| **Pereira, 2014 (14)** | 2002-2012 | patients that attend an ambulatory clinical analysis lab [Aveiro District] | 7,894 | 60.4 | ≈35 (age 0-5); 22.1 (age 6-15); 37.2 (age 16-25); ≈55 (age 26-35); ≈70 (age 36-45); ≈77 (age 46-55); 76.9 (age 56-65); ≈72 (age ≥66) |
| **Guerra, 2015 (15)** | 2010-2015 | Individuals age≥40 years who attended an International Travel Consultation [Lisbon District] | 271 | 73.1 | - |
| **ISN 2017 (16)** | 2015-2016 | All individuals resident in Portugal for at least 12 months, ≥2 years-old, that attend clinical analysis labs | 2,692 | 58.1 | 8.7 (age 2-4); 16.1 (age 5-9); 22.6 (age 10-14); 21.4 (age 15-19); 14.4 (age 20-29); 46.0 (age 30-44); 73.3 (age 45-54); 93.5 (age ≥55) |
| **Rocha, 2017 (17)** | 2012-2014 | Individuals ≥18 years traveling to international areas, that received medical care at Travel Clinic [Coimbra District] | 665 | 57.6 | 5.0 (age 18-25); 32.3 (age 26-30); 40.9 (age 31-35); 45.8 (age 36-40); 68.7 (age 41-45); 70.1 (age 46-50); 80.8 (age 51-55); 87.7 (age ≥56) |
| **Silva, 2018 (18)** | 2002-2012 | Individuals who attended an International Travel Consultation [Leiria District] | 2,001 | 73.8 | 50.8 (age 30-40); 80.8 (age 41-50); 94.8 (age 51-60); 97.1 (age ≥61) |

**References**


1. Lecour H, Ribeiro AT, Amaral I, Rodrigues MA. Prevalence of viral hepatitis markers in the population of Portugal. Bull World Health Organ. 1984;62(5):743-7.
2. Lecour H, Tomé-Ribeiro A, Amaral I, Rodrigues MA. Epidemiological aspects of acute viral hepatitis in Portugal. Infection. 1986;14(2):71-3
3. Leitao S, Santos RM, Santos JC, Ferreira R, Goncalves FN, Coutinho P, et al. Hepatitis A prevalence in rural and urban Portuguese populations. 1996;7:119-21.
4. Marinho RT, Valente AR, Ramalho FJ, de Moura MC. The changing epidemiological pattern of hepatitis A in Lisbon, Portugal. Eur J Gastroenterol Hepatol. 1997 Aug;9(8):795-7.
5. Macedo G, Ribeiro T. Hepatitis A: insights into new trends in epidemiology. Eur J Gastroenterol Hepatol. 1998;10(2):175.

6. Barros H, Oliveira F, Miranda H. A survey on hepatitis A in Portuguese children and adolescents. J Viral Hepat. 1999;6(3):249-53.
7. Lecour H, Santos I, Granjeira L, Candeisa J, Ramos J, J T. [Prevalência de marcadores da hepatite A e da hepatite E na população da região Norte de Portugal]. Arq Med. 1999;143:244-8.
8. Cunha I, Antunes H. [Prevalence of antibodies against hepatitis A virus in a population from northern Portugal]. Acta Med Port. 2001;14(5-6):479-82.
9. Antunes H, Macedo M, Estrada A. [Hepatitis A virus prevalence: Portuguese first results of low endemicity]. Acta Med Port. 2004;17(3):219-24.
10. DGO. Avaliação do Programa Nacional de Vacinação: 2º Inquérito Serológico Nacional Portugal Continental 2001-2002. Direcção Geral de Saúde; 2004.
11. Valadas E, Sousa S, F A. [Seroprevalência da hepatite A em infectados por vírus da imunodeficiência humana.] RPDI - Revista Portuguesa de Doenças Infecciosas. 2008;4:12-6.
12. Antunes H, Neiva F, Estrada A. Learn more in preventing infants' hepatitis A: The prevalence of hepatitis A virus antibody in Portuguese pregnant women population. J Pediatr Gastroenterol Nutr. 2009;48:E65-E6.
13. Speidel A, Malaba N, Marques R, Alves J, Veríssimo R, Valente C, et al. [Prevalência do Anticorpo contra o VHA numa população na Região Centro de Portugal]. Rev Port Doenças Inf. 2010;6:95-100.
14. Pereira S, Linhares I, Neves AF, Almeida A. Hepatitis A immunity in the District of Aveiro (Portugal): an eleven-year surveillance study (2002-2012). Viruses. 2014;6(3):1336-45.
15. Guerra AC, Aleixo MJ, Varela C, Remédios I, Nóbrega R, Lemos A, et al. [Rastreio serológico da hepatite A numa consulta do viajante–experiência do Hospital Garcia de Orta]. RPDI - Revista Portuguesa de Doenças Infecciosas. 2015;11:95-101.
16. INSA. Inquérito Serológico Nacional 2015-2016: Doenças Evitáveis por Vacinação. 2017.
17. Rocha S, Tejo S, Ferreira E, Trindade L, Rabadão E, Marques N, et al. Prevalence of Hepatitis A Virus Antibody in Portuguese Travelers: A New Paradigm. Acta Med Port. 2017;30(7-8):534-40.
18. Silva A, Passadouro R, Rodrigues R, Pascoal D, Soares F. [Imunidade para o vírus da hepatite A: vigilância de onze anos (2002-2012) numa consulta de medicina das viagens da região centro de Portugal.] RPDI - Revista Portuguesa de Doenças Infecciosas. 2018;14:7-12.

**Table S2.** Sociodemographic variables recoded and analysed in this study

| Variables | ISN 2001-2002 | ISN 2015-2016 | This study |
|---|---|---|---|
| **Education level** | | | |
| | Can neither read nor write | Not attended school | No formal instruction/ Basic (1st cycle) |
| | Primary Incomplete | - | |
| | Primary Complete | 1st Cycle/ Primary School | |
| | 2nd year (6th current) | 2nd Cycle/ 6th grade schooling/ Preparatory Cycle | Basic (2nd and 3rd cycle) |
| | 5th year (9th current) | 3rd Cycle/ 9th grade schooling/ 5th grade of high school | |
| | 7th grade (11th/12th actual) | Secondary school/ 12th grade/ High school complementary course | Secondary |
| | High School/ Polytechnic Course/University | University education (any degree) | Higher education |
| | Not applicable | - | Not applicable (on school age) |
| | Unknown | Unknown | Unknown |
| | Don't know | - | |
| **Country of birth** | | | |
| | Portugal | Portugal | Portugal |
| | Not collected | Argentina, Brazil, Venezuela | South America |
| | Not collected | Romania, Russia, Ukraine | Central and Eastern Europe |
| | Not collected | Germany, France, Luxembourg, Switzerland, The Netherlands, United Kingdom | Western Europe |
| | Not collected | Spain | Southern Europe |
| | Not collected | África do Sul, Angola, Cabo-Verde Guinea-Bissau, Mozambique, São Tomé | Africa |
| | Not collected | Australia, Canada, United States of America, Macau | Other |
| **Region of Residence** | | | |
| | Districts: | | NUTS II: |
| | Bragança; Braga; Porto; Viana do Castelo; Vila Real | | North |
| | Aveiro, Castelo Branco, Coimbra, Guarda, Leiria, Viseu | | Centre |
| | Lisbon, Santarem, Setubal | | Lisbon and Tagus Valley |
| | Beja, Evora, Portalegre | | Alentejo |
| | Faro | | Algarve |

**Table S3.** Comparison of samples characteristics between the two nationwide surveys (2001-2002 and 2015-2016), and with the Portuguese population in 2001 and 2015.

| | Nationwide sample tested | | Portuguese population | |
|---|---|---|---|---|
| | **2001-2002** | **2015-2016** | **2001** | **2015** |
| **Age groups (years), n (%)** | | | | |
| ≥2 - <30 | 820 (49.9) | 1287 (62.7) | 3710315 (35.7) | 2955827 (28.6) |
| 30 - 49 | 388 (23.6) | 377 (18.4) | 2962513 (28.5) | 2963595 (28.7) |
| ≥50 | 434 (26.4) | 388 (18.9) | 3494597 (33.6) | 4253959 (41.1) |
| **Sex, n (%)** | | | | |
| Male | 627 (38.2) | 1014 (49.4) | 5019374 (48.3) | 4901509 (47.4) |
| Female | 1015 (61.8) | 1038 (50.6) | 5375295 (51.7) | 5439821 (52.6) |
| **Region of residence, n (%)** | | | | |
| North | 514 (31.3) | 399 (19.4) | 3245279 (31.2) | 3179573 (30.7) |
| Centre | 435 (26.5) | 379 (18.5) | 2398424 (23.1) | 2275021 (22) |
| Lisbon and Tagus Valley | 553 (33.7) | 563 (27.4) | 3398721 (32.7) | 3529373 (34.1) |
| Alentejo | 79 (4.8) | 305 (14.9) | 460752 (4.4) | 413244 (4) |
| Algarve | 61 (3.7) | 406 (19.8) | 400937 (3.9) | 441929 (4.3) |
| | 1642 (100.0) | 2052 (100.0) | 10394669 (100.0) | 10341330 (100.0) |

**FIGURES**

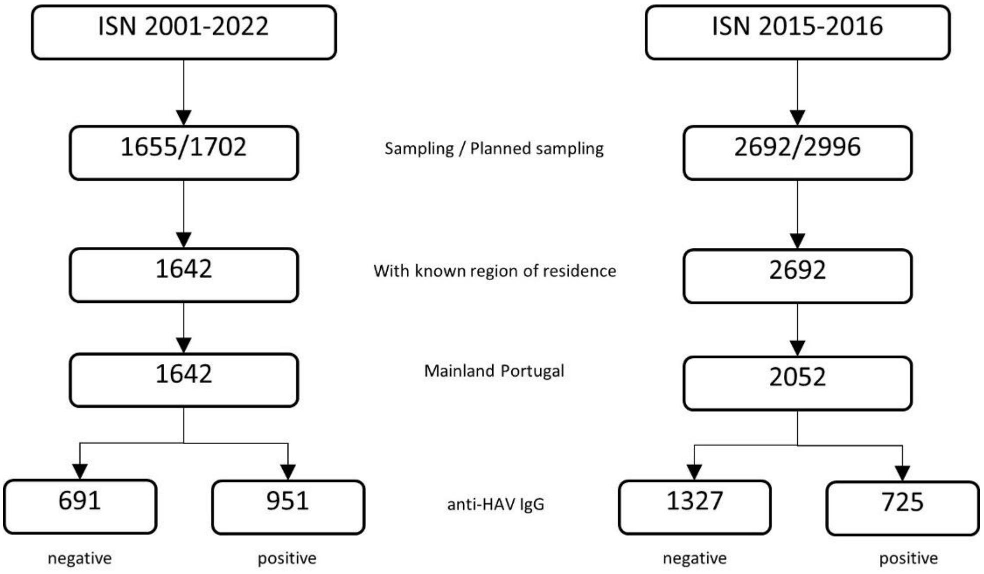


**Figure S1.** Summary of the data cleaning process to create the final study population.
